# Grocer Druggists

**Published:** 1904-06

**Authors:** 


### Grocer Druggists.

IN MANY districts more or less far removed from drug stores it has been the custom for grocers to keep for sale such drugs as may be termed household medicines, such as paregoric, camphor, Rochelle salts, ammonia and in many cases carbolic acid and oxalic acid. The attention of the state board of pharmacy was called to this fact some time ago and in order that there might be no quibble in correcting what was an evident danger, although unquestionably a convenience, the matter was submitted to Attorney-General Cunneen for his opinion as to the legality of the exposure for sale of the articles mentioned, among others, by the grocery men.

Mr. Cunneen has rendered an opinion at some length in which he views the matter dispassionately and in a logical manner. He decided that the sale by a grocer of any article coming under the head of drugs was illegal; that some of the articles mentioned in the communication from the pharmacists were used in the arts, such as oxalic acid, for example. Such articles may be kept for sale by grocers, but only for use in the arts and not to be disposed of in small quantities to families for household use. The opinion is one which may cause some inconvenience in neighborhoods where there is not a drug store, but it will definitely prevent the possibility of fatal error in dispensing by one who is neither qualified nor licensed to dispense poisons and drugs. The liability to error in the handling of oxalic acid and Rochelle salts is too well-known to need more than a passing reference. The dispensing of drugs should properly be confined to licentiates in pharmacy,—just where Mr. Cunneen's decision places it for good and all.

---

DEATHS from the bites of serpents are numerous in Asia and in Africa. Dr. S. Weir Mitchell, of Philadelphia, says *The Tribune,* for several years carried on researches of no small importance with regard to the venom of poisonous snakes, and announced finally that he had discovered no effective antidote. At the annual meeting of the Association of American Physicians, in Washington, Dr. Mitchell is said to have informed his medical associates that he had received a letter from Dr. Nōguchi, of Japan, who has been working at the Serum Institution, in Copenhagen, setting forth the discovery of a serum which will cure the sufferer from the bite of a rattlesnake. Will this antidote prove efficacious also against the venom of the cobra and other deadly serpents? If so, it will be of remarkable benefit to mankind, especially among the natives of India.

---

THE new board of regents, reduced and consolidated, held a meeting at the Capitol in Albany, April 26, 1904, at which Whitelaw Reid was elected chancellor and Saint Clair McKelway vice-chancellor of the university.

The board confirmed the following-named appointments by Commissioner Draper: First assistant commissioner, in charge of technical and professional education, Howard J. Rogers; second assistant commissioner, in charge of academies and high schools, Edward J. Goodwin; third assistant commissioner, in charge of primary education, Augustus S. Downing; director of libraries, Melvil Dewey. Each of the four has a salary of $5,000.

John M. Clarke was made director of science work at a salary of $3,600.

The following-named heads of administrative departments were appointed: accounts, William Mason, salary, $2,500; attendance, James D. Sullivan, $3,000; examinations, Charles F. Wheelock, $4,000; inspections, Frank H. Wood, $3,500; law, Edwin N. Holbrook, $3,500; records, Charles E. Fitch, $2,500; statistics, Hiram C. Case, $2,400.

---

A WOMAN, upon whose child Velpeau, the great French surgeon, had performed a most difficult operation, according to *The Argonaut,* called upon him, full of gratitude, and presented him with a pocketbook which she had embroidered with her own hands. Velpeau received the testimonial very crustily, saying that it was a beautiful pocketbook, and all that, but that his necessities demanded something more substantial. "My fee," he said, coldly, "is 5,000 francs." The woman very quietly opened the pocketbook, which contained ten one-thousand-franc notes, counted out five, and, politely handing them to Velpeau, retired.

---

Dr. G. C. MARTIN, instructor in paleontology at Johns Hopkins, has been appointed by the government as head of an expedition to Alaska next summer to investigate the recently discovered coal and oil fields, and to complete maps of the unknown portions of the peninsula.

---

ANTONIUS, the boy wonder, (aged 40) has, through a lawyer, recently filed notice that he would appeal to the Court of Appeals from the conviction of conspiring to defraud the public by a fake healing scheme. The appellate division recently affirmed the conviction. Weichers, the culprit's alleged real name, was sentenced to about a year in the penitentiary, but is out on bail.

---

IT SEEMS we were in error in stating in our May edition that New York City was about to witness the passing of the coroner. Mayor McClellan has seen fit to veto the bill for what seemed to him, we presume, good and sufficient reasons. In general, we favor the substitution of medical examiners for coroners, and shall consider the state at-large fortunate when it shall so provide by law.

There seems to be considerable opposition to the bill just vetoed in New York City, the *Post-Graduate,* among other journals, approving Mayor McClellan's action. However, the Mayor is in error when he says the medical examiner system has not worked satisfactory in other places or states. In Massachusetts, where it has been in vogue for some years, it meets approval, and in Erie county, where it has been in operation two years or more, it has proved a great improvement over the old system.

---

WE HAVE received recently a polite invitation to become a subscriber to the *Brooklyn Medical Journal,* accompanied by the usual "specimen copy." The invitation enumerates the qualities of the journal, among which it modestly says it is "the best medical journal published in New York State." This reminds us of the old story of the prisoner awaiting trial, who said to a visitor one morning,—one of his old associates,—"Bill, I'm going to be acquitted next week." "How d'ye know, Jim?" "Why, I've the best lawyer in the city to defend me, and he says he'll clear me." "Who is he?" "Parkinson." "Parkinson, why I never heard of him. How d'ye know he's the best lawyer in the city?" "Why,—because—why—be—*he admits it himself.*"

